# Movement detection thresholds reveal proprioceptive impairments in developmental dyslexia

**DOI:** 10.1038/s41598-020-79612-4

**Published:** 2021-01-11

**Authors:** Julie Laprevotte, Charalambos Papaxanthis, Sophie Saltarelli, Patrick Quercia, Jeremie Gaveau

**Affiliations:** 1 INSERM UMR1093-CAPS, Université Bourgogne Franche-Comté, UFR des Sciences du Sport, Dijon, France; 2 Centre de Formation Universitaire en Orthophonie, Université de Franche-Comté, UFR Sciences de La Santé, Besançon, France

**Keywords:** Neurological disorders, Cognitive neuroscience, Development of the nervous system, Learning and memory, Motor control, Peripheral nervous system, Sensorimotor processing, Sensory processing, Somatosensory system, Human behaviour, Paediatric research

## Abstract

Developmental dyslexia is associated with vision and hearing impairments. Whether these impairments are causes or comorbidities is controversial. Because both senses are heavily involved in reading, cognitive theories argue that sensory impairments are comorbidities that result from a lack of reading practice. Sensory theories instead argue that this is sensory impairments that cause reading disabilities. Here we test a discriminant prediction: whether sensory impairments in developmental dyslexia are restrained to reading-related senses or encompass other senses. Sensory theories predict that all senses are affected, whereas, according to the lack of reading practice argument, cognitive theories predict that only reading-related senses are affected. Using a robotic ergometer and fully automatized analyses, we tested proprioceptive acuity in seventeen dyslexic children and seventeen age-matched controls on a movement detection task. Compared to controls, dyslexics had higher and more variable detection thresholds. For the weakest proprioceptive stimuli, dyslexics were twice as long and twice as variable as controls. More, proprioceptive acuity strongly correlated with reading abilities, as measured by blind cognitive evaluations. These results unravel a new sensory impairment that cannot be attributed to a lack of reading practice, providing clear support to sensory theories of developmental dyslexia. Protocol registration: This protocol is part of the following registration, ClinicalTrials.gov Identifier: NCT03364010; December 6, 2017.

## Introduction

Developmental dyslexia (DD) is a specific learning disorder causing persistent failure to acquire efficient reading. It is the most prevalent neurodevelopmental disorder, affecting about 9% of children^1^, and it has profound lifelong consequences on academic and professional success^2^. Understanding the pathophysiological mechanisms of DD is paramount to advance this society's grand challenge.

Thousands of studies have investigated the pathophysiology of DD, thereby documenting various impairments and developing highly debated theories^3,4^. Although DD has long been considered a purely cognitive disorder, mounting evidence revealed that DD is associated with sensory impairments^5,6^, for a review see^7^. Yet, an outstanding question remains. Do sensory impairments cause DD? On one side, sensory theories argue that sensory deficiencies cause reading disabilities^4,7^. On the other side, cognitive theories argue that this is the lack of reading practice that causes sensory deficits^8,9^. Because DD is mostly associated with visual and hearing impairments—two senses heavily involved in reading—this long-standing controversy is still prominent.

According to sensory theories, troubles are supposed to encompass the whole sensory system, not only vision and hearing^4,7^. Cognitive theories, instead, would hypothesize that sensory troubles are restrained to reading-related senses. Results that support the generalization of sensory impairments in DD are scarce^10^. Testing a sensory function that is independent of reading practice would thus significantly advance the pathophysiology of DD. A positive result would undeniably support sensory theories and vice-versa.

Proprioception is an essential component of sensorimotor control^11,12^, and several studies have reported sensorimotor impairments in DD^13,14^. Here, we directly test proprioceptive acuity in dyslexic children and age-matched controls.

## Results

**Figure 1:**
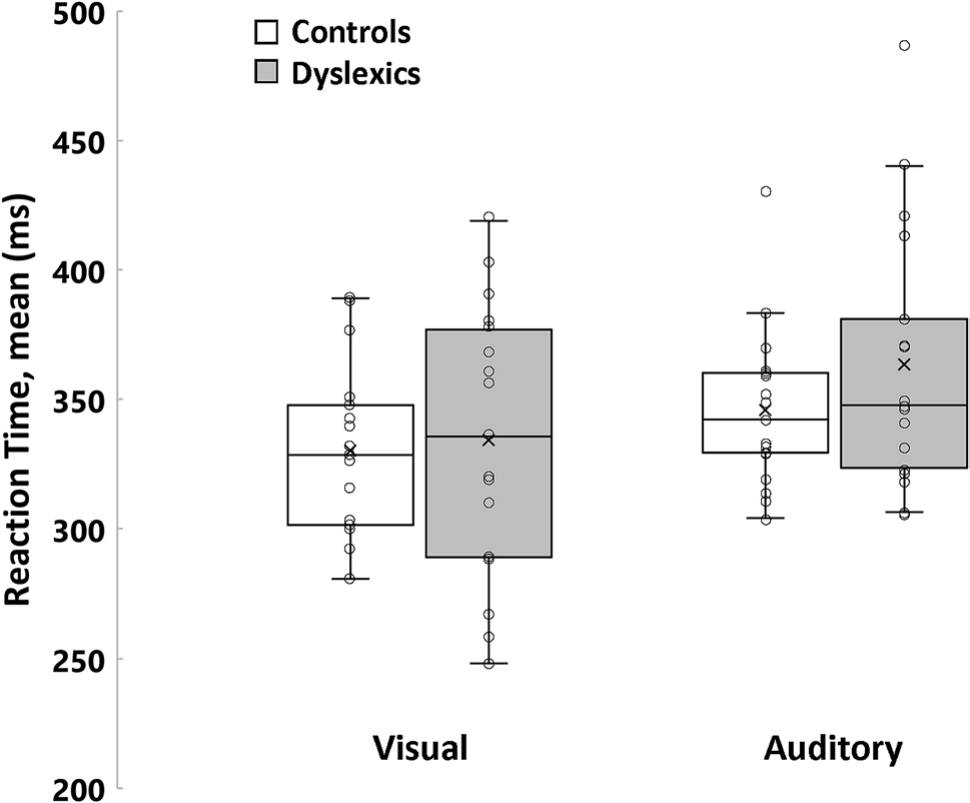
Mean Visual and Auditory Reaction Times. The box lines indicate the 25th, 50th, and 75th percentiles, and the whiskers extend to the 5th and 95th percentiles. Dots display individual values (n = 17 in each group), and the cross indicates the average.

To compare basic reaction time levels between groups, we measured simple reaction times to salient visual and auditory stimulus (See Methods and supplementary Table 1 for values). Figure 1 reveals similar reaction times in dyslexics and controls for both sensory modalities (visual: t(32) = 0.26, p = 0.79, d = 0.09 ; auditory: t(32) = 1.22, p = 0.23, d = 0.42). Since developmental dyslexia is associated with noisier behaviors^13,15^, we also compared variable reaction times—i.e., intra-participants variability—between Controls and Dyslexics. Variable visual and auditory reaction times were similar between groups (visual: t(32) = 0.19, p = 0.85, d = 0.19; auditory: t(32) = − 0.43, p = 0.67, d = 0.15). It is worth reminding that vision and hearing impairments have already been demonstrated in DD for more difficult sensory tasks^5,6,15^. Here, simple reaction time to salient stimulus cannot capture these fine impairments and only provide baseline results to help interpreting finer measurements in the proprioceptive task.

**Figure 2:**
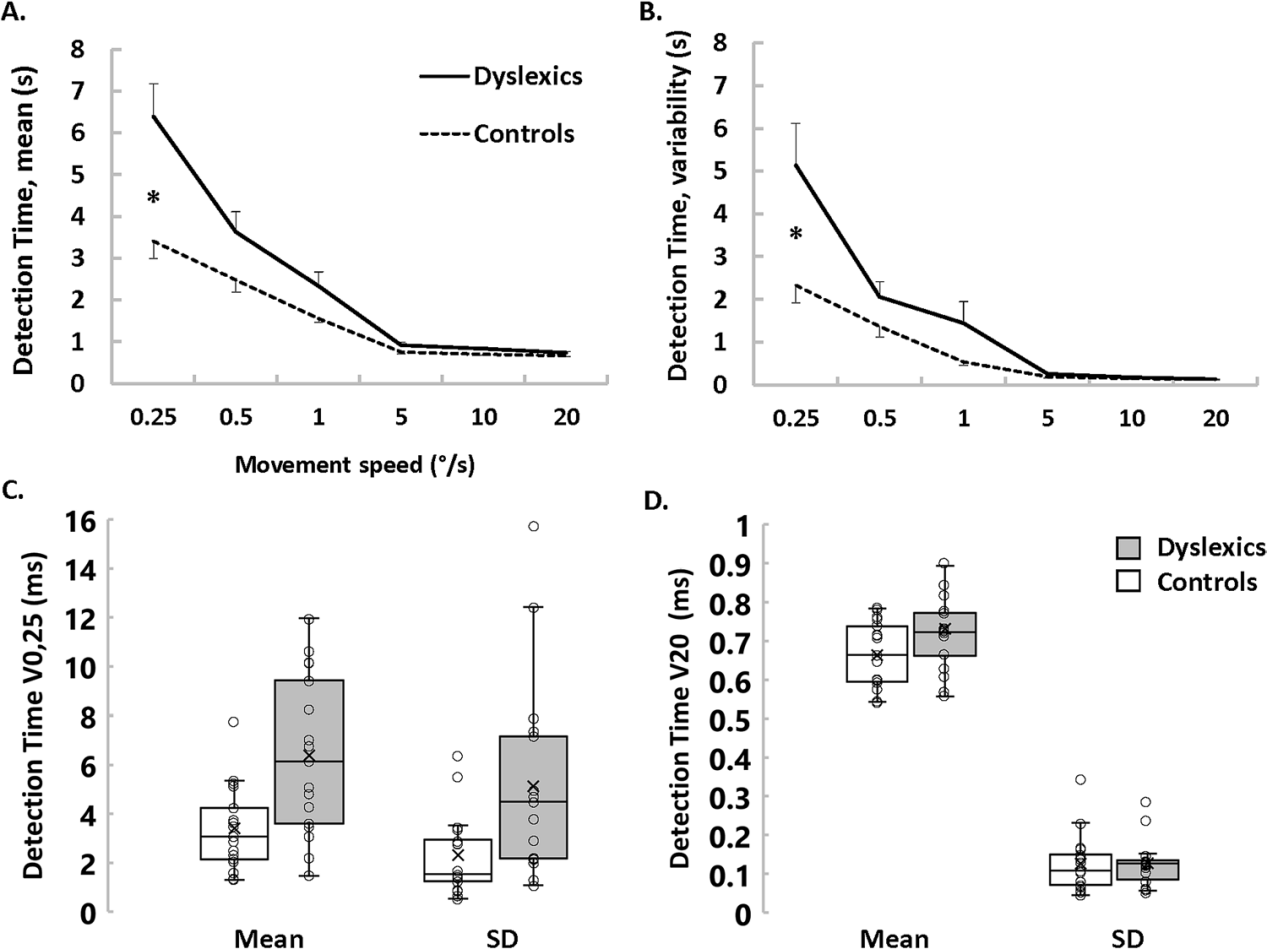
Proprioceptive detection times of passive motion at all tested speeds. (**A**) Mean detection time (± SE) for both groups. (**B**) Mean intra-participant variability of detection time (± SE) for both groups. (**C**) Box plots of mean and variable detection times at the slowest speed. The box lines indicate the 25th, 50th, and 75th percentiles, and the whiskers extend to the 5th and 95th percentiles. Dots display individual values (n = 17 in each group), and the cross indicates the average. (**D**) Box plots of mean and variable detection times at the highest speed. Same organization as panel (**C**).

We also used a simple reaction time task to compare proprioceptive acuity between dyslexics and controls. A robotic manipulandum passively rotated their elbow joint, and children pressed a trigger as soon as they felt the motion. The robot speed changed from trial to trial, thereby modulating the proprioceptive stimulation intensity; i.e., the signal to noise ratio. Proprioceptive impairments are known to cause longer and more variable reaction times for the weakest stimulations^16^. Figure 2A–B displays mean and variable proprioceptive detection times of the two groups for the six passive movement speeds (see supplementary Table 2 for values). Both Dyslexics and controls showed the well-known effect of stimulus intensity; i.e., mean and variable proprioceptive reaction times decreased when passive movement speeds increased^16,17^. It is striking, however, that the slope of these relationships differed between groups. Although dyslexics and controls exhibited similar values at higher speeds, dyslexics were slower and more variable than controls at slower speeds. At the slowest speed (0.25° s^−1^), dyslexics were even twice as long and twice as variable as controls. Out of seventeen dyslexics, twelve (71%) fell outside normality (95% confidence interval) according to their mean reaction time and ten (59%) according to their variable reaction time. ANOVA yielded strong *group* x *speed* interaction effects on mean [F(5,160) 9.60; *p* < 1e−6; η^2^ = 0.23] and on variable proprioceptive reaction times [F(5,160) 7.22; *p* = 4e−6; η^2^ = 0.18]. *Post-hoc* comparisons confirmed that dyslexics were longer (*p* = 2.7e−5, d = 1.14) and more variable (*p* = 1.6e−4, d = 0.92) than controls at the slowest speed (0.25° s^−1^). This echoes previous results showing noise-related sensorimotor impairments in DD; for a review see^4,15^.

Box-plots in Fig. 2C–D detail mean and variable reaction times for the slowest and the highest speed. Although results obtained at the slowest speed show clear group effects (Fig. 2C), results obtained at the highest speed demonstrate that dyslexics and controls responded equally quickly to proprioceptive stimulations (Fig. 2D). Also, comparing the first three to the last three trials did not reveal any effect in dyslexics nor in controls (t(16) = 9e−3; p = 0.99, *d* = 0.04) nor in controls (t(16) = 1.16; p = 0.26, *d* = 0.01). Thus, in both groups, the baseline response speed to proprioceptive stimuli was stable throughout the experiment. Furthermore, EMG activations of three major muscles around the elbow joint ensured that dyslexics and controls equally-well respected the instruction to keep their muscles relaxed during robotic manipulations (see supplementary Table 3).

**Figure 3:**
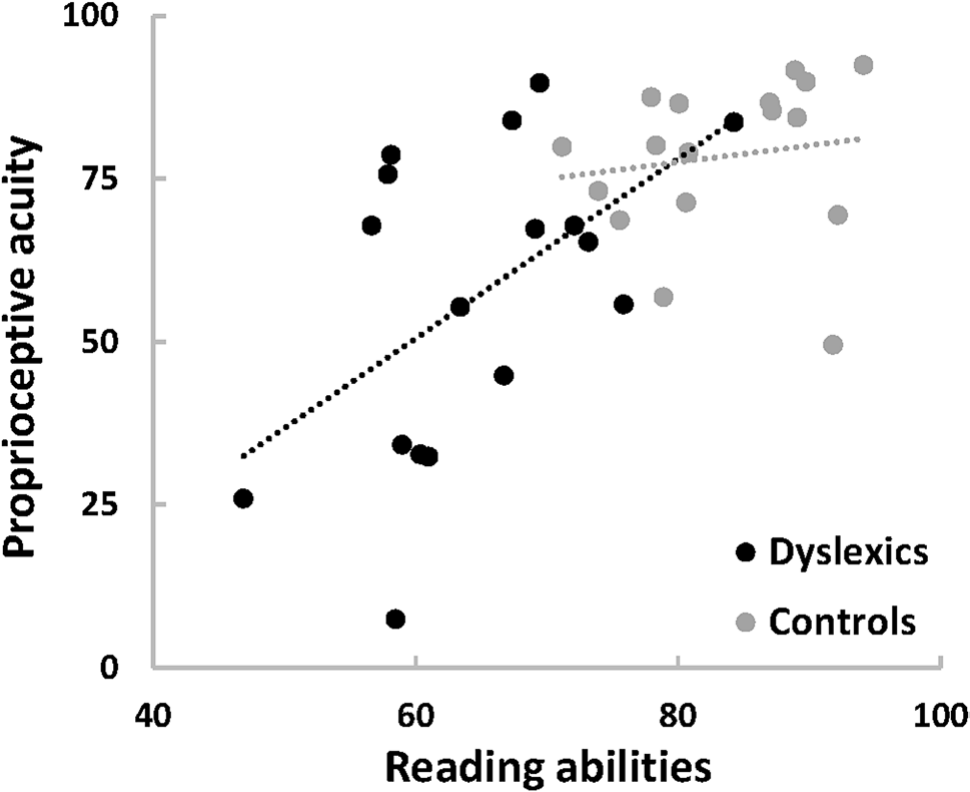
Correlation between proprioceptive acuity and reading abilities (n = 17 in each group). An index representing proprioceptive acuity (vertical axis) was computed as the normalized average of Mean and variable detection time at the slowest speed. An index representing reading abilities (horizontal axis) was computed as the normalized average of four clinical scores that are routinely used to diagnose developmental dyslexia (see Methods). Dashed lines depict linear regressions for each group.

Next, one wonders how much proprioceptive impairment can explain reading impairment. We computed Pearson's correlation coefficients between a proprioceptive acuity index and a reading ability index (see methods). Within these indexes, higher values mean better function. Figure 3 reveals a strong positive correlation between proprioceptive acuity and reading ability (Pearson R = 0.61, p = 1.3e−4). Importantly, this is not the result of a bimodal distribution of dyslexics' and controls' data. When pooled across groups, both indexes showed normal distribution (p > 0.2). When separating groups, a significant correlation existed in the dyslexic group (Pearson R = 0.52, p = 0.03) but not in the control group (Pearson R = 0.15, p = 0.57). No such correlation existed between the reading ability index and visual or auditory reaction times (see supplementary Table 4). Thus, although not all dyslexics show a strong proprioceptive impairment, the continuous distributions of proprioceptive and reading levels are tightly linked. More, proprioceptive indexes of the elbow and hip joints*—*tested in a subset of 12 participants (6 dyslexics and 6 controls) – were significantly correlated in dyslexics (Spearman R = 0.83, p = 0.04) but not in controls (Spearman R = 0.03, p = 0.96). Proprioceptive impairment in dyslexia likely generalizes to other joints.

## Discussion

The first level of interpretation of the present results concerns the understanding of the pathophysiological mechanisms of DD. Until now, sensory impairments in DD were mostly documented for vision and hearing^5,6,10^, for a review see^7^, making them prone to the criticism that they could result from a lack of reading practice^8,9^. Because reading does not solicit arm and hip proprioception, the lack of reading practice cannot explain the sizeable proprioceptive impairment revealed in this study. Instead, our results undeniably support the hypothesis that damaged neural mechanisms cause widespread sensory troubles in DD. The present results complement recent longitudinal and training evidence supporting sensory theories of developmental dyslexia^18–23^.

Several frameworks propose candidate neural structures and mechanisms whose impairment could explain sensory and sensorimotor troubles in DD. For example, the *Delayed neural commitment framework* proposes that abnormal temporalities in the development of distributed neural networks lead to reading and other learning disabilities^4^. More specific, the *Magnocellular theory* proposes that this is the impaired development of the Magnocellular system that causes poor temporal sensory processing, hence impeding efficient reading^7^. Impaired attentional processes have also been proposed to cause DD^24,25^. Particularly, feature-selective attention is known to influence noise-correlation in sensory cortices and may explain noise-exclusion deficit in DD^15,26,27^.

Research on other cognitive pathologies, such as autism and Alzheimer's dementia, suggests that testing sensorimotor functions allows more precocious diagnoses than testing cognitive functions^28,29^. Developmental studies of sensory and sensorimotor functions in DD have the potential to further our understanding of its etiology and to develop innovative remediations^4,8^. In addition to vision and hearing, future work shall also consider proprioception.

The second level of interpretation of the present results concerns the role played by proprioceptive impairments in DD. We found a strong correlation between a reading ability index and a proprioceptive acuity index. Proprioceptive acuity also significantly correlated with phonological processing alone (Pearson R = 0.45, p = 0.008). One may wonder whether proprioception could cause reading impairments itself. The motor theory of speech perception^30^ postulated that gesture recognition participates in the perception of phonological units. Several experiments then demonstrated that mirror neurons allow such gesture recognition^31,32^ and that gesture recognition—involving proprioceptive and tactile information—indeed participates in phonological perception^33,34^. Thus, proprioceptive impairment, along with vision and hearing impairments, may well participate in causing Developmental Dyslexia.

## Materials and methods

### Participants

Eighteen children with developmental dyslexia (7 girls; mean age: 10.92 ± 0.78 years) and seventeen age-matched typical readers (9 girls; mean age: 11.11 ± 0.85 years) participated in the study. We restricted the age for inclusion to 10–12 years because previous work has shown that proprioceptive function was roughly stable between these boundaries for a review see^35^. All were native French speakers, right-handed (Edinburgh Handedness Inventory; Oldfield, 1971), and normally integrated the school grade corresponding to their age (neither skipped nor doubled) without need for specific assistance. It is important to stress here that, in French schools, medical doctors screen children for cognitive disabilities two times before that age. Thus, although not ideal, this indicates that all children had a normal cognitive level. They also had normal or corrected-to-normal vision, normal hearing, and no history of neurological, proprioceptive, nor psychiatric disorders. Children and parents gave their free and informed written consent, and an ethics committee approved all experimental procedures (Comite de Protection des Personnes Ile de France VII, France; number 2017-A01547-46). All procedures were carried out according to local requirements and international norms (Declaration of Helsinki, 1964).

We recruited dyslexics via independent speech and occupational therapists. The diagnosis of developmental dyslexia was established by specialists (neuropsychologists, neuropediatricians, or speech therapists), using both inventories and testing procedures following the DSM-IV or DSM-5 guidelines. We recruited control children in municipal associations and schools. Neither control children nor their close family members had ever seen a medical doctor or related therapist for learning disorders.

### Clinical evaluations

We screened all children for attentional^36^ and motor abilities^37^. The aim was to exclude children at risk of Attention Deficit Hyperactivity Disorder and/or Developmental Coordination Disorder. We excluded one dyslexic boy from the study because of his positive result on the Conners test.

A speech therapist blindly evaluated reading abilities of all children with three clinical tests: (i) the Alouette Reading Test^38^, which is the French standardized assessment of reading skills; (ii) the "Phonological Process" part of NEPSY 2^39,40^, evaluating phonological segmentation skills; (iii) the Timé 3^41^, that evaluates words identification. As expected, controls outperformed dyslexics on all measures (Table 1). We performed inclusion procedures and all above-mentioned clinical evaluations in one session, and the experimental sensory testing described here-after in a second session. For each child, no more than three months separated these sessions.

**Table 1:** Clinical Scores of Dyslexic and Control children at the Alouette, Nepsy2, and Timé3 tests (see Methods).

Test items	Dyslexics N = 17	Controls N = 17	T-test
	Mean ± SD	Mean ± SD	*t (32); p; cohen’s d*
Reading Time (s)—*Alouette-R*	178.65 ± 2.67	140.71 ± 26.09	t = 5.96; 1.2e−7; 2.05
Number of words read—*Alouette-R*	181.47 ± 63.29	258.71 ± 12.89	t = − 4.93; 2.4e−5; 1.69
Number of errors—*Alouette-R*	16.82 ± 10.13	8.12 ± 5.01	t = 3.18; 3.3e−3; 1.09
Accuracy—*Alouette-R*	165.23 ± 65.66	250.41 ± 13.15	t = − 5.24; 2.8e−7; 1.80
Efficiency Index—*Alouette-R*	167.12 ± 68.06	332.80 ± 72.64	t = − 6.86; 9.1e−9; 2.35
Accuracy Index—*Alouette-R*	89.78 ± 7.50	96.82 ± 2.92	t = − 3.61; 1e−3; 1.30
Phonological Process—*NEPSY2*	27.42 ± 12.76	36.23 ± 2.41	t = − 2.79; 8.7e−3; 0.96
Words identification (months)—*Timé3*	29.06 ± 12.90	− 11.59 ± 21.88	t = 6.45; 2.9e−7; 2.26

As expected, controls outperformed dyslexics on all measures. Efficiency Index = (A × 180)/RT, where A stands for Accuracy and is the number of words correctly read (including self-corrections), 180 is the maximum time (in seconds) allowed for the test, and RT is the reading time. Accuracy Index: (A/NWR) × 100, where A stands for Accuracy, and NWR is the number of words read.

### Sensory testing

First, to evaluate response speed, children performed a classical visual and auditory simple reaction time test using salient stimuli^42^. The order of visual and auditory tasks was pseudo-randomized. Children sat in front of a monitor with their left index finger touching the computer space key and had to press it as soon as they saw (460 * 550 pixels, high contrast) or heard (900 Hz, 50 dB) the stimulus. Ten visual and ten auditory stimuli were presented with a random—two to five seconds—inter-trial delay. Before the test, children performed three practice trials per sensory condition.

After a 10 min break, we measured proprioceptive acuity using a well-known movement detection task^16,17^, which evaluates the capacity to detect the passive motion of a body–limb at various speeds. A robotic isokinetic dynamometer delivered proprioceptive stimulations (Biodex Medical Systems®, Shirley, NY, USA). Children sat on a chair and placed their right forearm on the dynamometer's resting device. We aligned the dynamometer's axis of rotation with the elbow flexion/extension axis of each child. The initial position of the arm was as follows: shoulder abducted between 70° and 85°, elbow flexed at 60° (0° was elbow extension), and forearm fully pronated^16,17^. Within this configuration, the dynamometer passively flexed the elbow in the horizontal plane. We instructed children to relax their muscles and to focus on detecting passive flexion. Children had to press a trigger, held in their left hand, as soon as they felt the motion. Here we call "proprioceptive reaction time", the time between the motion start and the trigger pressing. The dynamometer moved with six different angular speeds: 0.25, 0.5, 1, 5, 10 and 20°s^−1^. A total of seventy-two trials (12 trials per speed, in random order) were completed in six blocks separated by a two-minute rest time to avoid tiredness. Before the test, children performed two practice trials at each velocity (twelve trials). A subset of participants (six dyslexics and six controls) also performed the same proprioceptive evaluation protocol on the hip joint.

Children wore opaque goggles and noise reduction headphones (Bose, QC25) to prevent sight and hearing from influencing perceptual judgments. Because changes in muscle history can lead to errors in limb position sense, we controlled for muscle thixotropy by asking children to contract the muscles around their right elbow for one second before each passive movement^11,43^. Children were also required not to perform any vigorous motor or cognitive activity three hours before the tests. Children were authorized to take breaks at any time to prevent fatigue during the experiment.

We recorded surface electromyography (EMG) over the biceps brachii (BB), the brachio-radialis (BR), and the Triceps brachii long head (TBL) to control that muscles were relaxed during proprioceptive stimulations. We recorded EMG using silver-chloride surface electrodes (7 mm recording diameter, Ag–AgCl, 20 mm inter-pole distance). The skin was shaved and cleaned with alcohol. We synchronically recorded EMG signals with torque and angle using a Biopac system (Biopac MP150, Biopac System Inc, USA, gain = 1000, band-passed 1 Hz–5 kHz, sampled at 10 kHz).

### Data analysis

We computed all parameters using custom MATLAB programs (MathWorks).

Reaction time. We computed visual and auditory reaction times as the time-interval between the presentation of the stimulus and the children's response on the keyboard/trigger. For each child and condition, we computed the mean and the variable reaction time (standard deviation of trials).

Electromyography. We rectified, band-passed (30–300 Hz), and integrated (300 ms skipping window) EMG signals. Then, we computed the ratio of EMG before movement start (− 300 ms) divided by maximum EMG during movement, and we used this ratio to compare muscle activation/relaxation between groups.

### Statistics

All variables showed normal distribution (Kolmogorov–Smirnov test) and equivalent variance (Levene test). We performed group comparisons using independent two-tails t-tests (visual, auditory reaction times, and clinical results). We compared proprioceptive reaction times using repeated-measure ANOVA with a group (Controls vs. Dyslexics) and a speed factor (0.25°s^−1^, 0.5°s^−1^, 1°s^−1^, 5°s^−1^, 10°s^−1^ and 20°s^−1^), and post-hoc analyses (Scheffe) when appropriate. We also computed Pearson's correlation coefficients between reading abilities and elbow proprioceptive acuity. We computed a proprioceptive acuity index as the normalized average of mean and variable reaction times (at the slowest speed). We computed a reading ability index as the normalized average of four clinical scores classically used to diagnose DD in France (see Table 1): the Alouette Speed Index, the Alouette Accuracy index, the NEPSY2 result, and the Timé 3 result. We used Spearman correlation coefficients to compare arm and hip proprioceptive indexes.

## Ethics approval

The ethics committee Comite de Protection des Personnes Ile de France VII, France (2017-A01547-46) approved all experimental procedures.

## Consent to participate

Children and parents gave their free and informed written consent to participate.

## Consent for publication

Children and parents gave their free and informed written consent for results to be published.

## Supplementary information

Supplementary Information.

## Data Availability

Data files are available on request to the corresponding author.

## Code availability

Codes used for data analyses are available on request to the corresponding author.

## References

[1] Katusic SK, Colligan RC, Barbaresi WJ, Schaid DJ, Jacobsen SJ (2001). Incidence of reading disability in a population-based birth cohort, 1976–1982, Rochester, Minn. Mayo Clin. Proc..

[2] Boetsch EA, Green PA, Pennington BF (1996). Psychosocial correlates of dyslexia across the life span. Dev. Psychopathol..

[3] Démonet J-F, Taylor MJ, Chaix Y (2004). Developmental dyslexia. Lancet.

[4] Nicolson RI, Fawcett AJ (2019). Development of dyslexia: the delayed neural commitment framework. Front. Behav. Neurosci..

[5] Lovegrove WJ, Bowling A, Badcock D, Blackwood M (1980). Specific reading disability : differences in contrast sensitivity as a function of spatial frequency. Science.

[6] Kraus N (1996). Auditory neurophysiologic responses and discrimination deficits in children with learning problems. Science (80-.).

[7] Stein J (2019). The current status of the magnocellular theory of developmental dyslexia. Neuropsychologia.

[8] Goswami U (2015). Sensory theories of developmental dyslexia: three challenges for research. Nat. Rev. Neurosci..

[9] Ramus F (2003). Developmental dyslexia: specific phonological deficit or general sensorimotor dysfunction?. Neurobiology.

[10] Stoodley CJ, Talcott JB, Carter EL, Witton C, Stein JF (2000). Selective deficits of vibrotactile sensitivity in dyslexic readers. Neurosci. Lett..

[11] Proske U, Gandevia SC (2018). Kinesthetic senses. Compr. Physiol..

[12] Riemann BL, Lephart SM (2002). The sensorimotor system, part II: the role of proprioception in motor control and functional joint stability. J. Athl. Train..

[13] Pozzo T (2006). Static postural control in children with developmental dyslexia. Neurosci. Lett..

[14] Stoodley CJ, Fawcett AJ, Nicolson RI, Stein JF (2005). Impaired balancing ability in dyslexic children. Exp. brain Res..

[15] Sperling AJ, Lu ZL, Manis FR, Seidenberg MS (2005). Deficits in perceptual noise exclusion in developmental dyslexia. Nat. Neurosci..

[16] Konczak J, Krawczewski K, Tuite P, Maschke M (2007). The perception of passive motion in Parkinson's disease. J. Neurol..

[17] Li K-Y, Su W-J, Fu H-W, Pickett KA (2015). Kinesthetic deficit in children with developmental coordination disorder. Res. Dev. Disabil..

[18] Franceschini S, Bertoni S, Gianesini T, Gori S, Facoetti A (2017). A different vision of dyslexia: local precedence on global perception. Sci. Rep..

[19] Franceschini S (2013). Action video games make dyslexic children read better. Curr. Biol..

[20] Franceschini S, Gori S, Ruffino M, Pedrolli K, Facoetti A (2012). A causal link between visual spatial attention and reading acquisition. Curr. Biol..

[21] Carroll JM, Solity J, Shapiro LR (2016). Predicting dyslexia using prereading skills: the role of sensorimotor and cognitive abilities. J. Child Psychol. Psychiatry Allied Discip..

[22] Gori S, Seitz AR, Ronconi L, Franceschini S, Facoetti A (2016). Multiple Causal links between magnocellular-dorsal pathway deficit and developmental dyslexia. Cereb. Cortex.

[23] Bertoni S, Franceschini S, Ronconi L, Gori S, Facoetti A (2019). Is excessive visual crowding causally linked to developmental dyslexia?. Neuropsychologia.

[24] Bosse ML, Tainturier MJ, Valdois S (2007). Developmental dyslexia: the visual attention span deficit hypothesis. Cognition.

[25] Facoetti A (2010). Multisensory spatial attention deficits are predictive of phonological decoding skills in developmental dyslexia. J. Cogn. Neurosci..

[26] Cohen MR, Maunsell JH (2009). Attention improves performance primarily by reducing interneuronal correlations. Nat Neurosci..

[27] Downer JD, Rapone B, Verhein J, O'Connor KN, Sutter ML (2017). Feature-selective attention adaptively shifts noise correlations in primary auditory cortex. J. Neurosci..

[28] Teitelbaum P, Teitelbaum O, Nye J, Fryman J, Maurer RG (1998). Movement analysis in infancy may be useful for early diagnosis of autism. Proc. Natl. Acad. Sci. USA.

[29] Albers MW (2015). At the interface of sensory and motor dysfunctions and Alzheimer's disease. Alzheimer's Dement..

[30] Liberman AM, Mattingly TH (1985). The motor theory of speech perception revisited. Cognition.

[31] Fadiga L, Fogassi L, Pavesi G, Rizzolatti G (1995). Motor facilitation during action observation: a magnetic stimulation study. J. Neurophysiol..

[32] Gallese V, Fadiga L, Fogassi L, Rizzolatti G (1996). Action recognition in the premotor cortex. Brain.

[33] Moody J (1998). Proprioception and the McGurk effect. Science (80-.).

[34] Ito T, Tiede M, Ostry DJ (2009). Somatosensory function in speech perception. Proc. Natl. Acad. Sci. USA.

[35] Taylor S (2016). Does somatosensation change with age in children and adolescents? A systematic review. Child. Care. Health Dev..

[36] Conners, K. *Conners Rating Scales, Manual, 3rd Edition*. (2008).

[37] Marquet-Doléac, J., Soppelsa, R. & Albaret, J. *MABC-2—Batterie d’évaluation du mouvement chez l’enfant - 2nde édition. Adaptation Française*. (Pearson, 2016).

[38] Lefavrais, P. Alouette-R, Test d’analyse de la vitesse en lecture à partir d’un texte. Forme révisée. (2005).

[39] Korkman, M., Kirk, U. & Kemp, S. *NEPSY-II, Bilan Neuropsychologique de l’enfant, 2de édition.* (2012).

[40] Korkman, M., Kirk, U. & Kemp, S. *NEPSY II: clinical and interpretive manual. (2nd ed ed.)*. (2007).

[41] Ecalle, J. *Timé 3, test d’identification de mots écrits pour enfants de 7 à 15 ans*. (2006).

[42] Lum JAG, Ullman MT, Conti-Ramsden G (2013). Procedural learning is impaired in dyslexia: evidence from a meta-analysis of serial reaction time studies. Res. Dev. Disabil..

[43] Wise AK, Gregory JE, Proske U (1998). Detection of movements of the human forearm during and after co-contraction of muscle acting at the elbow joint. J. Physiol..

