# Supplementary material for: Movement detection thresholds reveal proprioceptive impairments in developmental dyslexia
Source: Sci Rep. 2021 Jan 11;11:299. doi: 10.1038/s41598-020-79612-4 (PMC7801726; doi:10.1038/s41598-020-79612-4)
Supplement: Supplementary file 1 — Supplementary Information. [file 41598_2020_79612_MOESM1_ESM.pdf]

Movement detection thresholds reveal proprioceptive impairments in developmental dyslexia

Julie Laprevotte[1], Charalambos Papaxanthis[1], Sophie Saltarelli[2], Patrick Quercia[1], Jeremie Gaveau[1*]

[1]INSERM UMR1093-CAPS, Université Bourgogne Franche-Comté, UFR des Sciences du Sport, Dijon, France.

[2]Centre de Formation Universitaire en Orthophonie, Université de Franche-Comté, UFR Sciences de la Santé, Besançon, France

*Correspondence to: jeremie.gaveau@u-bourgogne.fr

Supplementary Material

	**Dyslexic group N = 17**		**Control group N = 17**	
	**VRT**	**ART**	**VRT**	**ART**
**Mean (SD)**	334.28 (52.28)	363.41 (50.51)	331.81 (33.41)	345.87 (30.85)
**Variable (SD)**	55.95 (36.26)	47.49 (21.51)	53.83 (27.41)	52.00 (37.73)

Supplementary Table 1. Mean (SD) and variable (SD) reaction times (in ms) for vision and hearing. VRT = Visual Reaction Time, ART = Auditory Reaction Time.

Speed ($° s^{-1}$)	**0.25**	**0.50**	**1**	**5**	**10**	**20**
**Mean (SD)**						
Dyslexic group	6.39 (3.25)	3.62 (2.03)	2.32 (1.43)	0.91 (0.27)	0.83 (0.18)	0.73 (0.12)
Control group	3.41 (1.74)	2.48 (1.21)	1.55 (0.38)	0.75 (0.15)	0.70 (0.10)	0.66 (0.10)
**Variable (SD)**						
Dyslexic group	5.14 (4.01)	2.06 (1.44)	1.44 (2.08)	0.26 (0.15)	0.17 (0.09)	0.13 (0.06)
Control group	2.32 (1.71)	1.36 (1.12)	0.53 (0.30)	0.19 (0.16)	0.15 (0.07)	0.13 (0.08)

Supplementary Table 2. Mean (SD) and variable (SD) proprioceptive reaction times for dyslexics and controls at all speeds.

EMG Activities (mV)	Dyslexic group N = 17	Control group N = 17	T-test
	Mean (SD)	Mean (SD)	*t (32); p; cohen's d*
**Biceps brachii**	79.37 (27.81)	89.65 (8.58)	t= 1.46; 1.5e-1; 0.50
**Brachio-radialis**	87.65 (16.50)	92.47 (6.74)	t= 1.11; 2.7e-1; 0.38
**Triceps brachii long head**	81.05 (24.87)	88.57 (8.46)	t= 1.18; 2.5e-1; 0.50

Supplementary Table 3. Ratios of muscle activations before motion start (-300ms) divided by maximum activation during the robotic motion. Uncorrected T-test comparisons of EMG ratios showed no group effect. Dyslexics and controls equally-well respected the instruction to keep their muscles relaxed during robotic manipulations.

Pearson R		Read index		
		All n=34	Dys n=17	Cont n=17
Auditory	mean	-0,284	-0,306	0,033
	variable	0,011	-0,021	-0,122
	index	0,184	0,172	0,091
Visual	mean	-0,165	-0,389	0,174
	variable	-0,217	-0,317	-0,067
	index	0,159	0,376	0,002
Proprioception V0.25	mean	-0,547	-0,365	-0,108
	variable	-0,593	-0,573	-0,188
	index	0,610	0,516	0,148

Supplementary Table 4. Pearson R coefficients are presented for correlations computed between the reading ability index and reaction times. Correlations were computed merging the two groups (1st column) and at the group level (2nd and 3rd columns). Within each sensory modality, correlation with the read index was computed using mean reaction time, variable reaction time and an index combining both mean and variable reaction times (see methods).
